# Are Karst Rocky Desertification Areas Affected by Increasing Human Activity in Southern China? An Empirical Analysis from Nighttime Light Data

**DOI:** 10.3390/ijerph16214175

**Published:** 2019-10-29

**Authors:** Kaifang Shi, Qingyuan Yang, Yuanqing Li

**Affiliations:** 1 School of Geographical Sciences, State Cultivation Base of Eco-agriculture for Southwest Mountainous Land, Southwest University, Chongqing 400715, China; yizyang@swu.edu.cn; 2 Chongqing Jinfo Mountain Field Scientific Observation and Research Station for Kaster Ecosystem, School of Geographical Sciences, Southwest University, Chongqing 400715, China; 3 Chongqing Engineering Research Centre for Remote Sensing Big Data Application, School of Geographical Sciences, Southwest University, Chongqing 400715, China

**Keywords:** nighttime light data, human activities, karst rocky desertification, environmental impact, China

## Abstract

Due to remarkable socioeconomic development, an increasing number of karst rocky desertification areas have been severely affected by human activities in southern China. Effectively analyzing human activities in karst rocky desertification areas is a critical prerequisite for managing and restoring areas with tremendous negative impacts from desertification. At present, a timely and accurate way of quantifying the spatiotemporal variations of human activities in karst rocky desertification areas is still lacking. In this communication, we attempted to quantify human activities from the corrected Suomi National Polar-orbiting Partnership (NPP) Visible Infrared Imaging Radiometer Suite (VIIRS) Day/Night Band (DNB) nighttime light composite data from 2012 to 2018 based on statistical analysis. The results show that a significant increase of night lights could be clearly identified during the study period. The total nighttime lights (TL) related to severe karst rocky desertification (S) were particularly concentrated in Guizhou and Yunnan. The nighttime light intensity (LI) related to the S areas in Chongqing was the strongest due to its rapid socioeconomic development. The annual growth rate of nighttime lights (GL) has been slow or even negative in Guangdong because of its various karst rocky desertification restoration programs. This communication could provide an effective approach for quantifying human activities and provide useful information about where prompt attention is required for policy-making on the restoration of the karst rocky desertification areas.

## 1. Introduction

Karst rocky desertification has always been regarded as a very serious socioeconomic and environmental problem in the world [1]. The process of karst rocky desertification usually presents a pattern in which a karst area covered by vegetation and soil transforms into a rocky landscape that is almost devoid of vegetation and soil [2,3]. The expansion of karst rocky desertification would tremendously affect the ecologic, hydrologic, and soil environments and consequently lead to more land subsidence, landslides, droughts, and floods that threaten human sustainable development [4,5,6]. A crucial prerequisite for controlling and restoring karst rocky desertification is to determine the driving forces. It is generally known that karst rocky desertification is affected by many natural factors, such as slope, elevation, precipitation, temperature, and other factors [7]. Moreover, many studies have proven that anthropogenic factors (e.g., human activities) have gradually taken on the leading roles in karst rocky desertification [8,9,10]. Therefore, quantifying and analyzing human activities in karst rocky desertification areas is a critical prerequisite for managing and restoring the tremendous negative impacts of desertification.

The karst rocky desertification area in southern China is one of the world’s largest areas of karst geomorphology, and has experienced more extensive human activity since the beginning of the 21st century as compared to other karst areas [11]. Increasing economy, energy, and cultivation activities have led to more frequent human activities, causing soil erosion, agricultural production reduction, and tourism resource loss in karst rocky desertification areas [7,12]. For example, Xu et al. [9] found that the evolution of karst rocky desertification was negatively affected by human construction projects. Jiang et al. [7] indicated that huge population growth has forced people to conduct agriculture in many places with poor water availability and poor soil. The development of animal husbandry, such as goat and sheep grazing, could cause severe soil loss in the epikarst areas [7]. Extensive population growth has driven people to grow corn on steep hill slopes, resulting in severe soil erosion after a few years. Thus, accurately and effectively mapping and evaluating human activities is particularly crucial in karst rocky desertification areas in southern China.

Until now, many studies have analyzed human activities in karst rocky desertification areas at multiple scales (e.g., county, village, small town, or block scales), but few studies have analyzed those at the large regional scale [13]. Human activities are the comprehensive process of various economic and physical phenomena, but most of the studies have only explored one aspect of human activities, such as agricultural activities, deforestation, and infrastructure construction [14]. In addition, although some studies have attempted to explore human activities based on survey data and statistical data and have achieved very good results, few of them have been able to quantify the spatiotemporal variations of human activities due to the absence of spatial distributions [15]. Compared to traditional statistical or survey data, nighttime light data are unique, objective, and valuable data resources, and they have the advantages of providing efficient and accurate spatial data for observing human activity phenomena from a multiscale perspective [16,17]. A number of studies have proven that nighttime light data can reveal the spatial scope and intensity of human activities in relation to variables such as the gross domestic product (GDP) [18], population [19], carbon dioxide emissions [20], electric power consumption [21], housing vacancy rate [22], and urbanization [23,24]. Hence, the nighttime light data could be regarded as an effective and comprehensive proxy for the analysis of the spatiotemporal dynamics of human activities in karst rocky desertification areas.

Against these backgrounds, the objectives of this communication are to (1) quantify human activities from the nighttime light data at the large regional scale, and (2) evaluate spatiotemporal differences in human activities in karst rocky desertification areas in southern China. To achieve these goals, first, the nighttime light data were corrected. Then, the statistical analysis was used to quantify spatiotemporal differences in human activities. This study provides a suitable approach for mapping and analyzing human activities and for facilitating sustainable management and utilization of karst rocky desertification areas in southern China.

## 2. Methods 

### 2.1. Study Area

**Figure 1:**
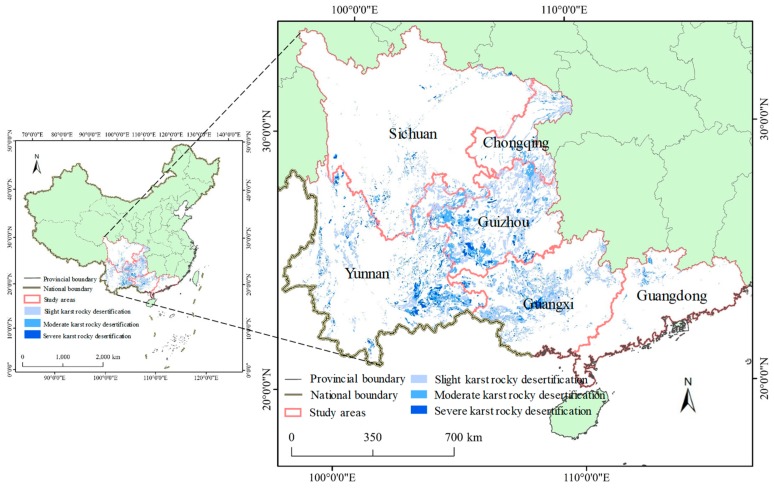
The spatial distribution of karst rocky desertification areas in southern China.

Six provincial administrative units in southern China, including Sichuan, Chongqing, Guizhou, Yunnan, Guangxi, and Guangdong, were selected as the study area (Figure 1) because China’s karst rocky desertification areas were mainly distributed in these provinces [7]. The study area has experienced rapid economic development, with GDP increasing from 2026 billion Yuan in 2000 to 19,455 billion Yuan in 2017 [25,26]. Meanwhile, the population increased without interruption from 324 million in 2000 to 358 million in 2017 [25,26]. Does rapid socioeconomic development aggravate karst rocky desertification in southern China? To answer this question, an important premise is to efficiently and accurately evaluate human activities related to karst rocky desertification areas in southern China.

### 2.2. Data Sources and Preprocessing

Three types of data were used in this communication, including nighttime light data, karst rocky desertification data, and administrative boundary data.

**Figure 2:**
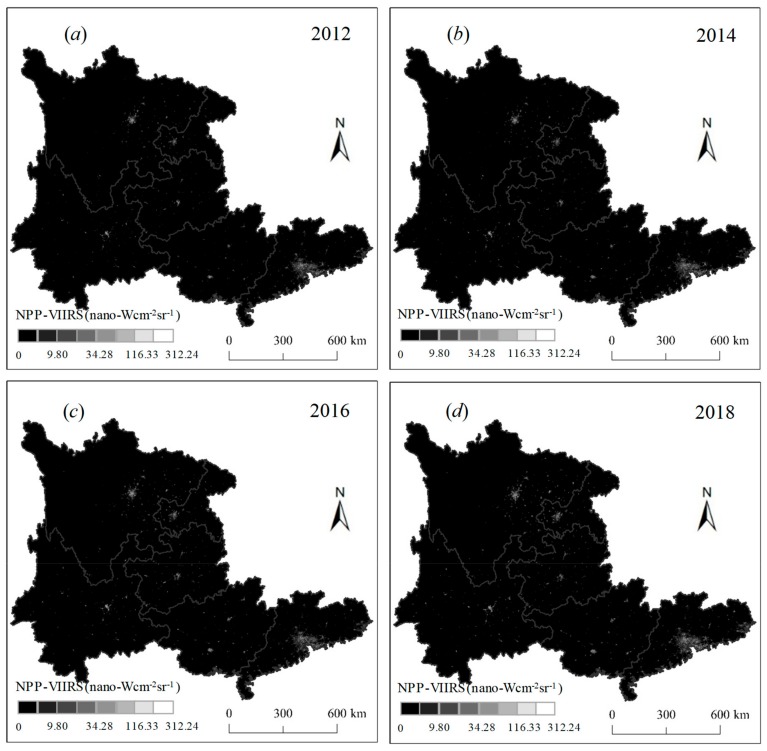
The corrected annually average National Polar-orbiting Partnership (NPP) Visible Infrared Imaging Radiometer Suite (VIIRS) data from 2012 to 2018—(**a**) 2012; (**b**) 2014; (**c**) 2016; (**d**) 2018.

The Visible Infrared Imaging Radiometer Suite (VIIRS) Day/Night Band (DNB) nighttime light data from the Suomi National Polar-orbiting Partnership (NPP) Satellite were used to map human activities. In comparison with the other nighttime light data, such as the Defense Meteorological Satellite Program’s Operational Linescan System (DMSP-OLS) nighttime light data, the NPP-VIIRS data have a much better spatial resolution. When compared with the Luojia 1-01 nighttime light data [27,28], the NPP-VIIRS data could also provide a long time series analysis. In this study, the 2012–2018 monthly NPP-VIIRS data were collected from the National Oceanic and Atmospheric Administration’s National Geophysical Data Center (NOAA/NGDC) (https://ngdc.noaa.gov/eog/viirs/download_dnb_composites.html). The data include all of the lights emitted by human beings at night and were produced in 15 arc-second segments (approximately 500 m). It should be noted that the original monthly NPP-VIIRS data are a preliminary product that have not removed bright noise surfaces, such as aurora, fires, boats, and other temporal lights [29]. Using the same method as the study of Shi et al. [18], a mask analysis and an eight-domain algorithm were developed to correct the monthly NPP-VIIRS data [18,30]. It is important to note that the maximum threshold setting was dependent on the nighttime lights of the airports in each province. To obtain the annual NPP-VIIRS data, a maximum value composition method and an average value composition method were compared in this communication. To demonstrate that the NPP-VIIRS data can effectively represent human activity, an effective way is to evaluate the correlation between the data and socioeconomic development, such as population and gross domestic product (GDP). This point has been proved by many previous studies [14,31]. When compared to the two datasets, we ultimately found that the average annual NPP-VIIRS data have a higher correlation with socioeconomic development in southern China. The average annual NPP-VIIRS data are shown in Figure 2.

The karst rocky desertification data in 2015 were obtained from the Institute of Geochemistry, Chinese Academy of Sciences (http://www.gyig.ac.cn/). The 2015 data present vector data through the interpretation of Landsat images. The karst rocky desertification data could be classified as three degrees: slight karst rocky desertification (L), moderate karst rocky desertification (M), and severe karst rocky desertification (S) [32]. The data have been proven to accurately represent the karst rocky desertification distribution in southern China by the study of Wang et al. [33].

The vector data of provincial and prefectural boundaries were downloaded from the National Geomatics Centre of China. 

All of the data were projected into the Albers equal-area conic projection, and the NPP-VIIRS data were resampled to a spatial resolution of 500 m before data bright noise correction.

### 2.3. Statistical Analysis

Three indicators were used to quantify human activities areas with karst rocky desertification: total nighttime lights (TL), nighttime light intensity (LI), and the annual growth rate of nighttime lights (GL), [14]. The formulas of TL and LI are specified as follows:

(1) $${\mathrm{TL}}_{j}^{c}=\overset{n}{\underset{i=1}{\sum }}{\mathrm{DN}}_{\mathrm{ij}}^{c}$$

(2) $${\mathrm{LI}}_{j}^{c}=\frac{{\mathrm{TL}}_{j}^{c}}{{\mathrm{TT}}_{j}^{c}}$$

where DN_ij_ represents the value of lighted pixel i in karst rocky desertification areas of j province, c represents the degree of karst rocky desertification, and TT represents the total number of lighted pixels.

According to He et al. [34] and Shi et al. [14], the GL was determined using the following formula:

(3) $${\mathrm{GL}}_{j}=\left({\left(\frac{{\mathrm{TL}}_{t_{2}}}{{\mathrm{TL}}_{t_{1}}}\right)}^{\frac{1}{t_{2}-t_{1}}}-1\right)\times 100\%$$

where TLt_1_ and TLt_2_ are the TL in year t_1_ and year t_2_, respectively.

## 3. Results and Discussion

### 3.1. Analysis of Spatial Distribution of Karst Rocky Desertification

**Figure 3:**
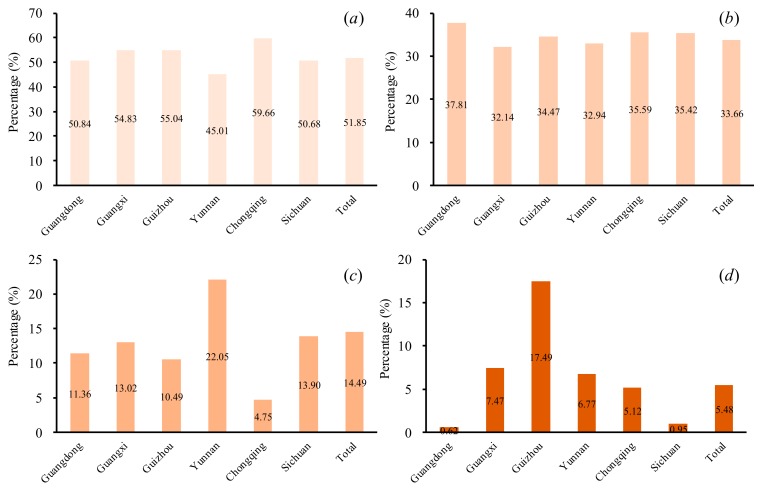
(**a**) The percentage of the total karst rocky desertification areas that have slight karst rocky desertification; (**b**) the percentage the total karst rocky desertification areas that have moderate karst rocky desertification areas; (**c**) the percentage the total karst rocky desertification areas that have severe karst rocky desertification areas; and (**d**) the percentage of the total karst rocky desertification areas in each administrative area.

From Figure 1, we found that the karst rocky desertification areas were widely distributed in southern China, accounting for 5.48% of the total area (Figure 3d). The karst areas with rocky desertification were generally dominant in Guizhou, Yunnan, Guangxi, and Chongqing but were less distributed in Guangzhou and Sichuan. Specifically, the karst rocky desertification areas comprised more than 5% of the total area in Guizhou (17.49%), Yunnan (6.77%), Guangxi (7.47%), and Chongqing (5.12%). However, Guangdong (0.62%) and Sichuan (0.95%) had relatively low percentages of karst rocky desertification areas of their total areas. 

A significant difference in the degree karst rocky desertification was identified at the provincial scale (Figure 3). In Guizhou, the slight karst rocky desertification (L) areas comprised 55.04% of the total karst rocky desertification (TA) areas (Figure 3a), with moderate karst rocky desertification (M) areas and severe karst rocky desertification (S) areas accounting for 34.47% and 10.09% of the TA areas, respectively (Figure 3b,c). In Chongqing, the L was the dominant type, accounting for 59.66% of the TA areas. It should be noted that the S areas composed 22.05% of the TA areas in Yunnan. In Guangdong, more than 400 km^2^ was related to the M areas, accounting for 37.81% of the TA areas. In total, the areas related to L accounted for 51.85% of the TA areas in the study area. The M areas accounted for 33.66% of the TA areas, and the S areas comprised 14.49% of the TA areas.

### 3.2. Analysis of the Nighttime Lights Related to Karst Rocky Desertification

**Figure 4:**
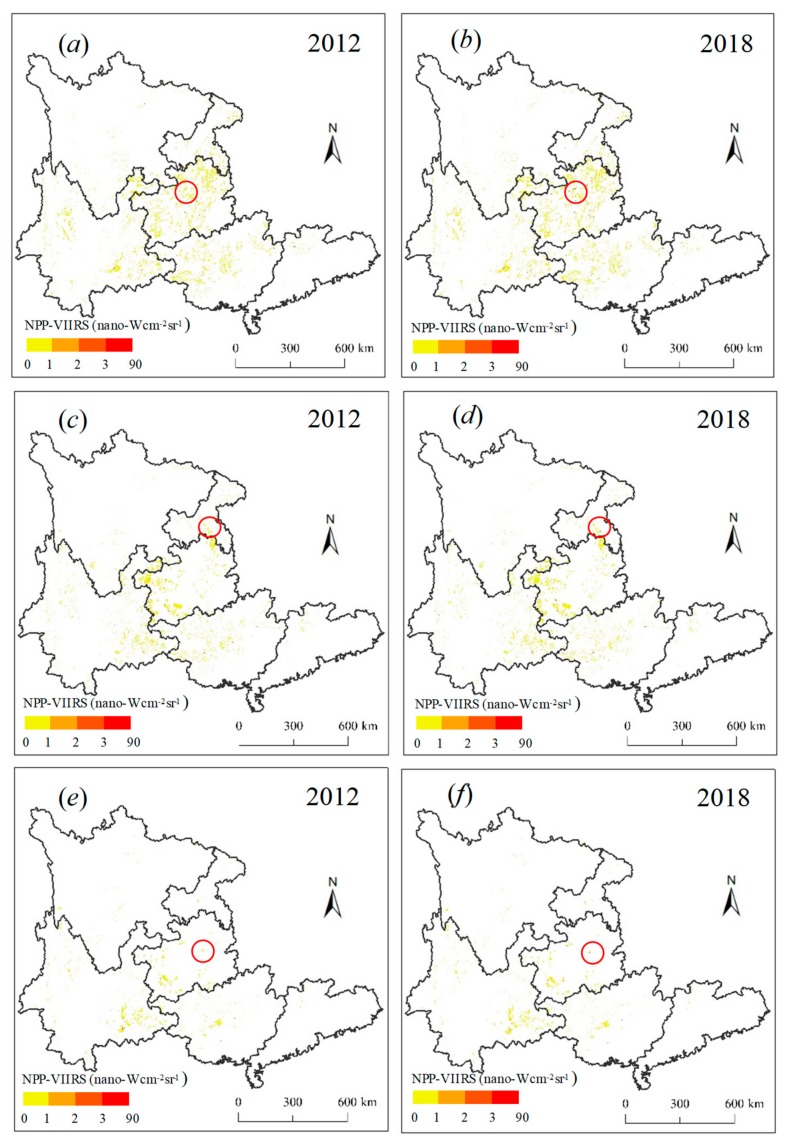
The corrected National Polar-orbiting Partnership (NPP) Visible Infrared Imaging Radiometer Suite (VIIRS) data related to karst rocky desertification from 2012 to 2018. Note: (**a**,**b**) The corrected NPP-VIIIRS data related to the L areas; (**c**,**d**) The corrected NPP-VIIIRS data related to the M areas; (**e**,**f**) The corrected NPP-VIIIRS data related to the S areas. L represents slight karst rocky desertification; M represents moderate karst rocky desertification; and S represents severe karst rocky desertification.

The spatiotemporal variation in nighttime lights related to karst rocky desertification in southern China from 2012 to 2018 are modeled in Figure 4. Significant increases of nighttime lights could be clearly identified during the study period. The TL in L areas increased rapidly from 16,433 nano-Wcm^−2^sr^−1^ in 2012 to 30,087 nano-Wcm^−2^sr^−1^ in 2018, with an average value of 22,366 nano-Wcm^−2^sr^−1^ (Table 1). The TL in L areas in Guizhou experienced the most rapid growth, with values from 6369 nano-Wcm^−2^sr^−1^ in 2012 to 13,591 nano-Wcm^−2^sr^−1^ in 2018. This is attributed to the many L areas located in Guizhou that have been undergoing rapid socioeconomic development. The TL related to M was widely distributed in Guangxi, Guizhou, and Yunnan. Yunnan had the highest TL growth in the M areas, with values from 3860 nano-Wcm^−2^sr^−1^ in 2012 to 5873 nano-Wcm^−2^sr^−1^ in 2018. The TL related to S was particularly concentrated in Guizhou and Yunnan. Although the TL related to S in Guizhou was not as high as that in Yunnan, the growth value was faster than that of Yunnan. It can be inferred that human activities would have tremendous impacts on the S areas in Guizhou due to the lack of sufficient land for development. In total, due to the implementation of the Western Development Strategy, increasing numbers of human activities are transforming the area from China’s eastern region to its southwest region (e.g., Guizhou, Yunnan, Guangxi, Chongqing, and Sichuan) [13,14], resulting in increasing industrial activities, infrastructure construction, tourism development, and deforestation, which could lead to the concentration of many nighttime lights concentrating in the karst rocky desertification areas.

By quantifying the LI over the different the degree of karst rocky desertification in southern China (Table 2), we could find that the high LI was mainly located in the S areas, followed by the M, and L areas, with average values of 0.17 nano-Wcm^−2^sr^−1^, 0.14 nano-Wcm^−2^sr^−1^, and 0.13 nano-Wcm^−2^sr^−1^ respectively. The results are fairly unexpected, and warn us to pay more attention to human activity impact on the S areas. The LI related to L has the highest strength in Guangdong, and presents a low value in Yunnan and Chongqing. Similarly, for the LI related to the M areas, the values increased from 0.14 nano-Wcm^−2^sr^−1^ in 2012 to 0.21 nano-Wcm^−2^sr^−1^ in 2018 in Guangdong. Thus, we inferred that human activities had a significant impact on the L and M areas in Guangdong than those of other provinces. Moreover, it was found that the LI related to the S areas in Chongqing maintained the highest strength, with the values from 0.36 nano-Wcm^−2^sr^−1^ in 2012 to 0.39 nano-Wcm^−2^sr^−1^ in 2018. This is because that Chongqing has been experiencing rapid growth of GDP and population since 1997. Due to the lack of adequate space for development, more and more intense anthropogenic disturbances, including mining, overgrazing, inappropriate farming practices, and other intensive uses have severe impacts on the S areas in Chongqing [33].

**Figure 5:**
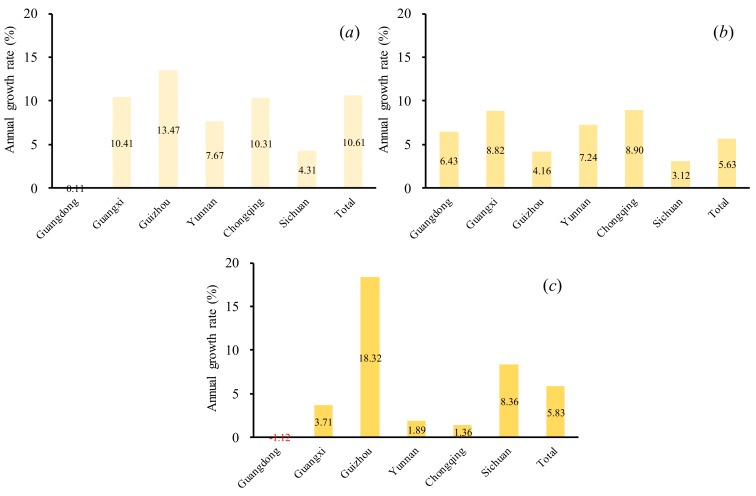
(**a**) The L annual growth rate from 2012 to 2018; (**b**) The M annual growth rate from 2012 to 2018; (**c**) The S annual growth rate from 2012 to 2018. Note: L represents slight karst rocky desertification; M represents moderate karst rocky desertification; and S represents severe karst rocky desertification.

We further evaluated the GL at the provincial scale (Figure 5). The high GL was also found to be mainly distributed in the L areas (10.61%), followed by the S (5.83%) and M areas (5.63%). For the L areas, the GL in Guizhou, Guangxi, and Chongqing looked much higher than in Guangdong, Sichuan, and Yunnan. For the M areas, there was still a relatively even distribution of the GL for each province. For the S areas, the GL experienced rapid growth from 2012 to 2018 in Guizhou, with a value of 18.32%. An interesting finding is that the GL has experienced slow or even negative growth in Guangdong. This might be a result of the various karst rocky desertification restoration programs [9]. A series of rocky desertification restorations strategies have also been developed and implemented in Guangdong. Because Guangdong is the richest province in China, it has spent a lot of money on ecological environment restoration, such as ecological migration and forest conservation.

## 4. Conclusions

In this study, we attempted to reveal human activities in karst rocky desertification areas of southern China from 2012 to 2018 using the NPP-VIIRS nighttime light data. First, the NPP-VIIRS data were corrected in this study. Then, the statistical analysis was applied to evaluate spatiotemporal variations in nighttime lights in the different degrees of karst rocky desertification areas. The results clearly show that the karst rocky desertification areas were widely distributed in southern China, accounting for 5.48% of the total area. The TL related to S was particularly concentrated in Guizhou and Yunnan. It was found that the LI related to the S areas in Chongqing was the strongest due to the rapid growth of the region’s GDP and population since 1997. This study could provide a new approach for the timely quantification and evaluation of human activities in karst rocky desertification areas that have fragile ecological environments. Due to the limitation of the length of communication, we do not provide any suggestions for karst rocky desertification reduction. In future studies, we will continue to evaluate the mechanisms of human activities related to karst rocky desertification from multi-source remote sensing data, and provide a series of suggestions for strategies. In addition, because it is hard to distinguish the difference in human activity lights based on the nighttime light data, we will attempt to describe what type of human activity is presented in this region that emits nighttime lights, and which of these activities are unsuitable for desertification. Future studies will also attempt to quantify nighttime lights over different land cover types in karst rocky desertification areas.

## Figures and Tables

**Table 1:** The total nighttime lights related to karst rocky desertification in southern China.

DD	Name	Year							Mean
		2012	2013	2014	2015	2016	2017	2018	
		(nano-Wcm^−2^sr^−1^)							
L	Guangdong	739	721	820	642	573	709	744	707
	Guangxi	4554	5051	5037	5678	5991	7518	8253	6012
	Guizhou	6369	8761	8566	7826	10,175	14,398	13,591	9955
	Yunnan	3530	4031	3458	3922	3375	5160	5498	4139
	Chongqing	784	985	809	770	1085	1542	1413	1055
	Sichuan	456	574	484	442	415	523	588	497
	Total	16,433	20,123	19,174	19,281	21,613	29,850	30,087	22,366
M	Guangdong	231	281	239	218	204	284	336	256
	Guangxi	2604	2657	2758	2997	3219	4023	4322	3226
	Guizhou	4350	4725	4659	4494	5064	6,025	5556	4982
	Yunnan	3860	4294	4083	4420	4037	5371	5873	4563
	Chongqing	433	568	424	465	490	731	723	548
	Sichuan	469	793	474	393	378	461	565	505
	Total	13,960	15,332	14,652	15,002	15,408	18,912	19,393	16,094
S	Guangdong	253	246	224	189	163	211	237	218
	Guangxi	1361	1580	1445	1281	1474	1756	1694	1513
	Guizhou	1201	1688	1510	1798	2938	3859	3295	2327
	Yunnan	4142	4082	3739	3756	3262	4211	4634	3975
	Chongqing	284	276	231	207	279	322	308	272
	Sichuan	31	38	35	34	38	51	50	40
	Total	7273	7911	7183	7265	8155	10,409	10,218	8345

Note: DD represents the degree of karst rocky desertification; L represents slight karst rocky desertification areas; M represents moderate karst rocky desertification areas; and S represents severe karst rocky desertification areas.

**Table 2:** The nighttime light intensity related to karst rocky desertification in southern China.

DD	Name	Year							Mean
		2012	2013	2014	2015	2016	2017	2018	
		(nano-Wcm^−2^sr^−1^)							
L	Guangdong	0.33	0.32	0.37	0.29	0.26	0.32	0.34	0.32
	Guangxi	0.12	0.13	0.13	0.15	0.15	0.19	0.21	0.15
	Guizhou	0.09	0.13	0.13	0.12	0.15	0.21	0.20	0.15
	Yunnan	0.07	0.08	0.07	0.08	0.07	0.11	0.11	0.08
	Chongqing	0.08	0.10	0.08	0.08	0.11	0.15	0.14	0.11
	Sichuan	0.05	0.06	0.05	0.05	0.04	0.05	0.06	0.05
	Total	0.09	0.11	0.11	0.11	0.12	0.17	0.17	0.13
M	Guangdong	0.14	0.17	0.15	0.13	0.13	0.17	0.21	0.16
	Guangxi	0.11	0.12	0.12	0.13	0.14	0.18	0.19	0.14
	Guizhou	0.10	0.11	0.11	0.10	0.12	0.14	0.13	0.12
	Yunnan	0.11	0.13	0.12	0.13	0.12	0.16	0.17	0.13
	Chongqing	0.07	0.10	0.07	0.08	0.08	0.12	0.12	0.09
	Sichuan	0.07	0.12	0.07	0.06	0.06	0.07	0.09	0.08
	Total	0.12	0.13	0.13	0.13	0.14	0.17	0.17	0.14
S	Guangdong	0.32	0.31	0.28	0.24	0.20	0.26	0.30	0.27
	Guangxi	0.14	0.16	0.15	0.13	0.15	0.18	0.17	0.15
	Guizhou	0.09	0.13	0.12	0.14	0.23	0.30	0.26	0.18
	Yunnan	0.18	0.18	0.16	0.16	0.14	0.18	0.20	0.17
	Chongqing	0.36	0.35	0.29	0.26	0.36	0.41	0.39	0.35
	Sichuan	0.01	0.01	0.01	0.01	0.01	0.02	0.02	0.01
	Total	0.15	0.16	0.14	0.15	0.16	0.21	0.20	0.17

Note: DD represents the degree of karst rocky desertification; L represents slight karst rocky desertification areas; M represents moderate karst rocky desertification areas; S represents severe karst rocky desertification areas.
